# Towards the clinical translation of optogenetic skeletal muscle stimulation

**DOI:** 10.1007/s00424-020-02387-0

**Published:** 2020-05-15

**Authors:** Lili A. Gundelach, Marc A. Hüser, Dirk Beutner, Patrick Ruther, Tobias Bruegmann

**Affiliations:** 1 Institute of Cardiovascular Physiology, University Medical Center, Göttingen, Germany; 2 Department of Otorhinolaryngology, Head and Neck Surgery, University Medical Center, Göttingen, Germany; 3 Microsystem Materials Laboratory, Department of Microsystems Engineering (IMTEK), University of Freiburg, Freiburg, Germany; 4 BrainLinks-BrainTools Cluster of Excellence at the University of Freiburg, Freiburg, Germany; 5 DZHK e.V. (German Center for Cardiovascular Research), Partner Site Göttingen, Göttingen, Germany

**Keywords:** Skeletal muscle, Electrical stimulation, Optogenetic stimulation, Optical implants, Gene transfer, Immune response

## Abstract

Paralysis is a frequent phenomenon in many diseases, and to date, only functional electrical stimulation (FES) mediated via the innervating nerve can be employed to restore skeletal muscle function in patients. Despite recent progress, FES has several technical limitations and significant side effects. Optogenetic stimulation has been proposed as an alternative, as it may circumvent some of the disadvantages of FES enabling cell type–specific, spatially and temporally precise stimulation of cells expressing light-gated ion channels, commonly Channelrhodopsin2. Two distinct approaches for the restoration of skeletal muscle function with optogenetics have been demonstrated: indirect optogenetic stimulation through the innervating nerve similar to FES and direct optogenetic stimulation of the skeletal muscle. Although both approaches show great promise, both have their limitations and there are several general hurdles that need to be overcome for their translation into clinics. These include successful gene transfer, sustained optogenetic protein expression, and the creation of optically active implantable devices. Herein, a comprehensive summary of the underlying mechanisms of electrical and optogenetic approaches is provided. With this knowledge in mind, we substantiate a detailed discussion of the advantages and limitations of each method. Furthermore, the obstacles in the way of clinical translation of optogenetic stimulation are discussed, and suggestions on how they could be overcome are provided. Finally, four specific examples of pathologies demanding novel therapeutic measures are discussed with a focus on the likelihood of direct versus indirect optogenetic stimulation.

## Introduction

Paralysis is the general term used to describe a class of diseases characterized by loss of strength and control of skeletal muscle contractility. Over five million patients are affected by paralysis in the United States alone [6]. In most cases, this is not due to a problem with the muscles themselves but caused by diminished function of the neuronal input somewhere along the chain of nerve cells from the motoric cortex to the neuromuscular junction. The underlying diseases can be distinguished based on the anatomical classification of the upper and lower motor neurons. While all diseases of the brain will primarily affect the upper motor neurons, amyotrophic lateral sclerosis affects both upper and lower motor neurons, whereas spinal muscular atrophy or poliomyelitis and polyneuropathies are classified examples of lower motor neuron syndromes. Furthermore, several diseases can affect the neuromuscular junction, for example, myasthenia gravis and Lambert Eaton syndrome.

To date, research to restore skeletal muscle function in paralyzed muscles has primarily been focused on functional electrical stimulation (FES). Recently, optogenetic stimulation of skeletal muscle was proposed either by direct stimulation of the skeletal muscle fibers or indirectly by exciting the innervating nerves. This review first describes the underlying mechanisms of each approach and their status quo. Building upon these, we discuss in detail the requirements and the pros and cons of electrical versus optogenetic stimulation, as well as those of indirect versus direct stimulation. In addition, potential technical solutions for both stimulation modalities are discussed and critically compared. This discussion is substantiated by considering four diseases and their specific requirements for new therapeutic measures.

### Physiological activation of skeletal muscle

Skeletal muscles consist of myofibers oriented parallel to the macroscopic muscle, which form, in the direction of force generation, a complex structure with nerves, vessels, and connective tissue [81]. Importantly, all myofibers are electrically insulated from each other, allowing for their separate activation by single motor neurons and thus fine-tuned control of the generated muscle force. For this purpose, each motor neuron innervates only a certain amount of muscle fibers, referred to as a motor unit. In small motor units, one motor neuron innervates a few muscle fibers, which are mainly type I. Type I muscle fibers contain high amounts of myoglobin, contract slower and with low force development but are fatigue resistant. This explains why small motor neurons are activated first and especially in actions, which require fine-tuned control or have to be sustained over prolonged time periods. In contrast, large motor units typically activate type IIa and IIx fibers. These fast muscle fiber types contract faster and generate higher forces but are more prone to fatigue. Thus, larger motor units are recruited later on and/or for tasks which require maximal force but only for shorter time periods. This physiological recruitment order from the smallest (weakest) to the largest (strongest) motor units can be explained by the intrinsic properties of the motor neurons known as the “size-principle” [50]: Small motor units also have smaller motor neurons with small axonal diameter and less myelination compared with larger motor neurons from large motor units. In consequence, small motor neurons have a higher membrane input resistance and lower membrane capacity and require thus less dendritic input to get depolarized above the excitation threshold to become activated. However, once an action potential is evoked, the excitation spread is slower in small motor neurons due to less myelination. During prolonged phases of submaximal voluntary continuous contractions, the recruitment of motor neurons alternates as the initially activated ones become refractory and the others have lower activation thresholds [30]. On the other hand, during more maximal activation patterns, more motor neurons become activated at once, and local refractoriness of each motor neuron gains importance for example in the development of fatigue.

### Electrical stimulation of skeletal muscle

For any external stimulation of skeletal muscle, electrical insulation between the muscle fibers results in the need for individual stimulation of each muscle fiber or each motor neuron, as well as concurrent stimulation of all motor units to induce maximum force. Thus, direct electrical stimulation of the muscle fibers requires a large amount of energy [28, 53, 120], which leads to the generation of toxic gases [94], as well as the co-activation of nociceptive nerves, cutaneous mechanoreceptors, and adjacent muscles. Hence, direct electrical stimulation elicits painful sensations and non-specific movements, which makes it unsuitable for clinical use [53]. Importantly, recruitment of muscle fibers depends primarily on the position of the muscle fiber within the electrical field and not so much on its intrinsic properties. Due to the resultant equal activation of fast and slow fibers [18], fatigue development upon direct electrical stimulation mainly depends on the fiber composition of each muscle group.

Alternatively, skeletal muscles can be activated via indirect electrical stimulation of the innervating nerve. This is referred to as FES which can be performed at any location along the innervating motoric nerve or within the muscle, stimulating the presynaptic nerve endings. Due to their high input resistance, motor neurons can be activated with 200 times less energy compared with skeletal myofibers [53], which leads to reduced side effects. However, afferent sensory nerves can still be affected, especially in sensitive regions like the face or pharynx [104]. Unfortunately, motor unit recruitment is unphysiological, as larger motor units most commonly become activated before small motor units. This is believed to be due to their diameter and myelination, which defines the intracellular resistance and capacity, respectively, which is also reflected by the differences in the space constant [85, 121]. This rather unphysiological recruitment order leads to an aggravated fatigue development, especially during high force generation. Potential clinical applications in general are to facilitate training and muscle re-strengthening in injured patients [78, 143], or to restore skeletal muscle function after spinal cord injuries [142].

Because of its mechanistic principles, FES is effective if one motor nerve is innervating one muscle group performing one specific task. An example of this is phrenic nerve stimulation to improve respiration [84] with the ultimate aim to ameliorate side effects of passive long-term ventilation or aid recovery after lung transplantation [147]. Furthermore, the therapeutic benefit of electrical hypoglossal nerve stimulation to prevent relaxation of the tongue and subsequent closure of the airway has been demonstrated in patients suffering from obstructive sleep apnea [145]. When the stimulated nerve innervates several muscle groups, FES of the motor nerve itself cannot be performed in a muscle specific manner. Hence, the physiological concerted movement of hands or legs during precisely coordinated movements will be impaired. Regardless, FES using intramuscular electrodes can accomplish multinary hand-to-mouth activities after spinal cord injury [138]. However, the limits of what can be achieved in terms of more complex tasks, for example, handwriting, still have to be elucidated.

Importantly, FES requires intact motoric nerve function and cannot be used in cases of peripheral nerve dysfunction or diseases affecting the neuromuscular junctions, such as amyotrophic lateral sclerosis or myasthenia gravis [22].

### Optogenetic stimulation

Optogenetic techniques rely on genetic modification to enable light-elicited control of cellular processes via photosensitive proteins. Genetic overexpression enables cell type–specific stimulation, and the use of light as acute physical stimulus provides a level of spatial and temporal control that is impossible to attain using traditional electrical or pharmacological strategies. The incentive for optogenetics came from neurobiological research, as precise, cell-specific stimulation is vital for the understanding of brain function [21, 163]. The first attempt at an optogenetic system required the co-expression of three proteins from the Drosophila eye in neurons enabling optical control of neuronal activity [163]. However, the requirement for the overexpression of three proteins and the system’s low temporal kinetics prompted the search for a single component, directly light-responsive alternative.

Channelrhodopsin2 (ChR2), a light-sensitive, non-selective cation channel from *Chlamydomonas reinhardtii* was the first protein utilized as a single-component optogenetic tool. In mammalian cells, light-induced opening of ChR2’s pore leads to inward currents of monovalent cations, which depolarizes the cell membrane. Shortly after the discovery of ChR2, the feasibility to genetically introduce this protein to neurons and evoke neuronal action potentials by illumination ex vivo and in vivo was demonstrated by several groups [21, 56, 79]. Light-induced, ChR2-dependent muscle contractions were first demonstrated in *Caenorhabditis elegans* [105]. Ever since, new channelrhodopsin variants (ChR) have been created by inserting amino acid mutations in ChR2, finding new natural ChR in other species or creating chimera between these and ChR2. As a result, researchers can choose between a myriad of different ChR with distinct biophysical properties in terms of wavelength specificity, light sensitivity, current amplitudes, and on and off kinetics [92]. The idea of optogenetic therapeutic approaches emerged when Bi et al. [17] demonstrated that inner retinal neurons of blind mice can be used to restore the ability to react to light. This approach is now being tested in ongoing phase I/II clinical trials (NCT 02556736, ClinicalTrials.gov). Over the recent years, optogenetic techniques have gained increasing importance in basic research, especially in the field of neuroscience, and several approaches with future clinical potential have been described. These include brain implants to treat Parkinson’s disease [44], epilepsy [109], peripheral nerve stimulation to prevent chronic pain perception [57], or the restoration of urinary bladder function [97], as well as an optical stimulation of the cochlea [59]. The feasibility of optogenetic stimulation of the heart has also been demonstrated in several studies [23, 24, 26, 107, 152] with the highest translational potential for the treatment of cardiac arrhythmias [123].

For the restoration of skeletal muscle function, two different approaches have been proposed: indirect optogenetic stimulation through the innervating nerve or direct optogenetic stimulation of ChR2-expressing skeletal muscle.

### Indirect optogenetic stimulation

**Fig. 1:**
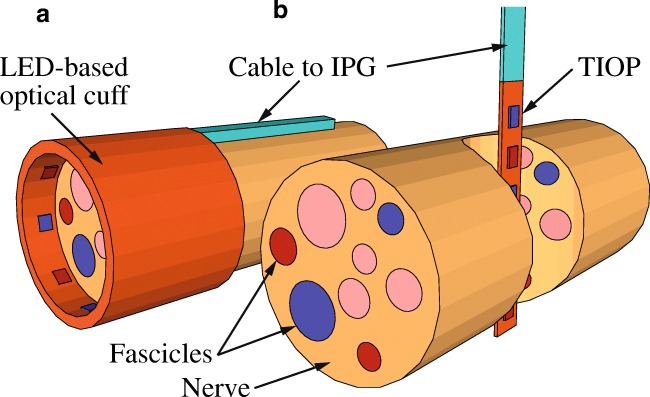
Illumination of motor neurons. Schematic of **a** an optical cuff implant and **b** a TIOP device used to interact with a peripheral nerve. The reflective coating of the optical cuff is not illustrated in the schematic. Color-coded fascicles either express two different ChR (blue and red) or are not AAV transduced (rose)

The first light-induced contraction of skeletal muscles was demonstrated by illumination of ChR2-expressing neurons in the motor cortex, which triggered movement of the whiskers [5] and locomotion in freely moving mice [45]. Soon afterwards, it was reported that optogenetic stimulation of the phrenic nucleus and spinal respiratory interneurons restored movement of the diaphragm and was able to restore breathing in rats after spinal cord injury [1]. Currently, the term “indirect optogenetic stimulation of skeletal muscles” is most commonly used to refer to the illumination of ChR2-expressing peripheral nerves. This was first demonstrated in transgenic animals [83] and later progressed to wild type mice utilizing adeno-associated viruses (AAV) [148]. AAV encoding optogenetic proteins can be injected systematically or locally into the target muscle. In the case of skeletal muscle, it is known that following local injection, the commonly used variant AAV 2.6 migrates retrogradely from the target muscle to the innervating nerve [149]. Consequently, only the motor neurons innervating this specific muscle group will express ChR2 and react to light stimulation of the whole nerve, which might also innervate various other muscle groups. Hence, a region of the nerve can be selected where the movement of the nerve-accompanying tissue is minimal to stimulate the specific target muscle group (Fig. 1a, b). Since the selective stimulation of two different ChR—one responding to blue light and the other two red light—appears feasible [39], this approach could be used to stimulate two different muscle groups. Activation of more than two muscle groups would require novel ChR with significantly UV- or infrared wavelength-shifted light sensitivity. Currently, it is difficult to envision the design of such variants, and thus indirect optogenetic stimulation appears to be limited to two distinct muscle groups or functions. Hence, plantarflexion and dorsalextension of the lower limb might be possible with this approach, however, it is not suitable for the restoration of the function of more complex systems like the forearm.

The use of red-shifted ChR, such as Chrimson [68] or ReaChR [82] allow optical stimulation via transdermal illumination because red light is absorbed by tissues to a lesser extent and thus penetrates much deeper than blue light. Such transdermal illumination is intriguing for basic research and especially in small animals bypassing the required implantation of an optical device, as well as challenges with respect to its energy supply. The translational prospect, however, can be questioned due to practical reasons as well as the risk of possible interactions with surrounding light. On the other hand, stimulation using optical nerve cuffs still allows the use of blue light as the diameters of the target nerves are normally within the range of a few millimeters.

One advantage of indirect optogenetic stimulation over FES lies in its higher similarity to the physiological recruitment order of motor units, as demonstrated by higher latency from stimulation to contraction, as well as slower contraction at low light intensities. The more physiological recruitment of motor units may underlie the decreased fatigue development upon indirect optogenetic stimulation compared with FES [83]. It has recently been suggested that lower fatigue development could also be due to the photo-kinetic behavior of the optogenetic system [141]. Similar to voluntary continuous contractions, the recruited motor neurons alternate during submaximal optogenetic stimulation. However, the behavior observed by Srinivasan et al. (2018) could also be explained by a more effective depolarization via ChR2 to higher levels of the membrane potential and prolonged stimulation periods. This suggests that optogenetic depolarization is more likely to overcome the local refractoriness with higher excitation thresholds of the motor neurons, especially at supramaximal stimulations [14, 49]. As a result, indirect stimulation of the plantar-flexor-group was fatigue-resistant for up to ~ 20 minutes, whereas the electrical stimulation remained at a similar level for only 20 s [83]. However, longer time periods, as well as sustained indirect optogenetic stimulation of skeletal muscle, have not been investigated so far. It is important to consider the side effects of illumination per se, as well as chronic ChR stimulation and the possible impact of these on nerve function [118].

### Direct optogenetic stimulation

Direct optogenetic stimulation of skeletal muscle was first demonstrated in *Caenorhabditis elegans* [105]. In mammals, direct stimulation of skeletal muscles was initially carried out in vitro in immortalized myoblasts from mice (C2C12 cells). Pulsed illumination of ChR2 expressing C2C12 myotubes was shown to result in a homogenous depolarization of the cell membrane inducing contractions [8]. This elegant proof-of-concept study was used later to investigate the propagation of membrane depolarization along the sarcolemma surface [131], enhance the maturation of C2C12 myotubes [9], and to improve movements of robotic actuators [115, 122].

Direct optogenetic stimulation of intact skeletal muscle tissue has been initially described in two individual studies published in 2015. In the first study [25], ChR2 expression was driven in transgenic mice by the chicken β-actin (CAG) promoter, a universal promoter highly active in myocytes. Light-induced contractions were examined in vitro in isolated fibers of the flexor digitorum brevis muscle and in intact, explanted soleus muscles. In the intact soleus muscles, the twitch contraction amplitude could be precisely controlled by varying light intensities. Approximately 84% of the maximal tetanic force obtained during electrical field stimulation could be generated in the soleus muscle by light stimulation, whereas there appeared to be no difference in muscle fatigue between optical and electrical stimulation during direct supramaximal stimulation [25].

The used ChR2 H134R shows generally a slow off-kinetic with approximately 20 ms and is thus restricted to efficient stimulation frequencies below 50 Hz. In comparison, the maximum effective stimulation rate for electrical stimulation is > 100 Hz, which explains the lower efficiency of optogenetic stimulation. Furthermore, blue light is highly absorbed by tissues. Consequently, recently developed red-shifted Chrimson-variants with fast off-kinetics [86] might exhibit a more optimal performance.

In the second study [87], the Cre/LoxP system was utilized in mice to trigger ChR2 expression in skeletal muscles using the skeletal muscle lineage-specific Sim1 promoter. Light-induced contractile responses were detected in vivo in intact hind limbs [87]. Importantly, repetitive optogenetic stimulation over several days could attenuate atrophy generation in denervated muscle fibers [87] which proves the feasibility and positive effects of direct optogenetic stimulation over several days.

However, it is important to note that both publications report optogenetic stimulation of soleus muscles which consist mainly of slow type muscle fibers [126, 127]. Induction of tetanic contractions by direct optogenetic stimulation in muscles consisting mainly of fast muscle fibers has not yet been demonstrated. Consequently, no reliable estimate of stimulation efficiency and fatigue development in mixed or fast muscle fibers can be made. Further investigation of these fibers is therefore vital. In theory, using specific promoters may also allow expression of optogenetic proteins specifically in slow or fast muscle fiber types in the future. Specific expression in slow fibers would reduce fatigue development.

**Fig. 2:**
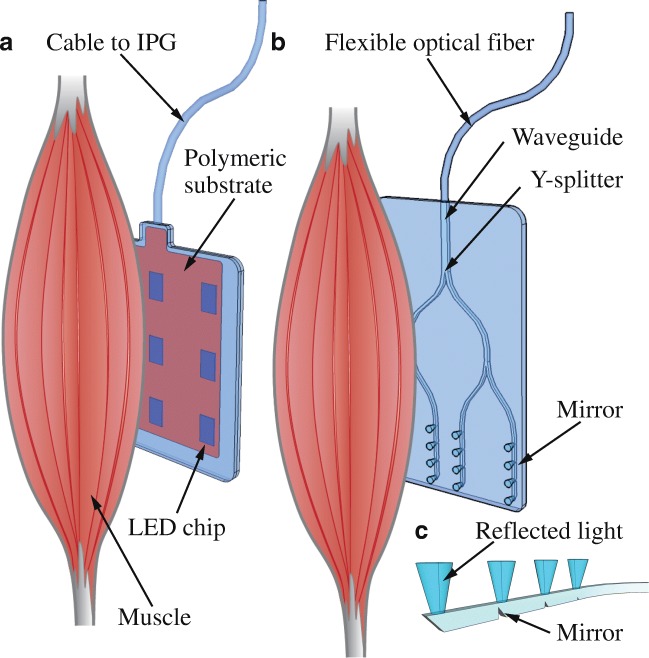
Illumination of skeletal muscles. Conceptual drawing of **a** a 2D LED array on a polymeric substrate encapsulated in silicone rubber or **b** a polymeric waveguide array with integrated mirrors and Y-splitters used for direct optical stimulation of a skeletal muscle. **c** Light reflected at 45° mirrors integrated into the waveguide structure of B

For direct optogenetic stimulation, illumination of the whole muscle is required in order to induce maximum force due to the anatomy and physiology of skeletal muscle tissue. Hence, optical implants capable of global illumination are needed and light must penetrate all layers of the muscle. In principle, this can be envisioned by an array of light-emitting diodes (LEDs, Fig. 2a) or a polymeric substrate with integrated waveguides (Fig. 2b) comprised of mirrors reflecting light perpendicularly to the plane of the optical probe (Fig. 2c). Importantly, such illumination is targeted specifically to numerous muscle groups and allows thus restoring several functions in parallel.

In conclusion, global illumination of skeletal muscles expressing ChR enables uniform depolarization of the whole myofiber with fine-grain control of its level and duration. Considering this, and the observed reduction of pathophysiological changes upon direct stimulation after denervation, it is likely that the function of paralyzed skeletal muscle could be restored even following severe peripheral nerve injuries and in case of peripheral nerve pathologies, or diseases affecting the neuromuscular junction such as amyotrophic lateral sclerosis and myasthenia gravis. However, in regions with large muscles exhibiting significant movement upon illumination, direct optogenetic stimulation of the muscle tissue is more challenging, as the implantable light devices have to accommodate for the high level of muscle movement, and the illumination fields would have to be very large. Furthermore, a reproducible, effective method of gene transfer is necessary to enable the therapeutic application of optogenetic approaches.

### The path towards clinical applications

Translation of optogenetic stimulation faces various obstacles which have to be overcome step by step in each application and be carefully weighted against the possible benefit for the patients. In this part, we will focus separately on the required gene transfer, the possible immune response against the gene transfer and ChR2, as well as the design of optically active implantable device (oAIMDs).

### Gene transfer

Therapeutic applications of optogenetic stimulation require sufficient gene delivery into the desired cells and tissue. This has the potential to confer a great level of controllability and specificity, as optogenetic protein expression can be restricted to a subset of cells in a promoter-dependent manner. Consequently, pain-free stimulation is possible because nociceptive fibers and cutaneous mechanoreceptors will not be co-activated like in the case of FES [71]. For example, optogenetic proteins can be specifically expressed in motor neurons or skeletal muscles. However, optogenetic expression also necessitates either genetic manipulation of the patient’s cells or transplantation of genetically modified cells derived from pluripotent cells, such as embryonic stem cells or induced pluripotent stem cells [27]. The clinical feasibility of the latter approach is an ongoing debate for safety reasons [72, 87, 134], as well as rather low efficacy. A more promising approach is the use of AAV. These vectors are derived from viruses, which are non-enveloped and comprise single-stranded DNA. Being dependoparvoviruses, they belong to the *Parvoviridae* family. AAV can efficiently transduce vertebral tissue and are not associated with any obvious clinical pathology [32]. Natural AAV are capable of site-specific integration into the host genome. However, the sequences required for integration have been removed from recombinant AAV used in gene therapy [35]. These vectors persist in an episomal form and are diluted by cell division in proliferating cells. It is therefore important to consider the proliferation rate of the targeted cell types when using AAV. Neurons have very limited ability to proliferate, and skeletal muscle is a low-turnover tissue under normal circumstances. However, skeletal muscle tissue is capable of extensive regeneration upon injury due to the presence of satellite cells. As AAV do not efficiently target muscle satellite cells [7], it is possible that transgene expression loss may occur over time.

Interestingly, different AAV serotypes show varying degrees of tropism towards different tissues [139]. This enables targeting of desired tissues even upon systemic virus delivery, which may even lead in the future to specific transduction of skeletal muscles or motorneurons.

Recombinant AAV have already been used in > 100 gene therapy clinical trials and their therapeutic potential has been confirmed in several genetic diseases, including muscular dystrophies [19]. Furthermore, the feasibility of AAV-based ChR2 delivery to skeletal muscle tissue has already been demonstrated in proof-of-concept experiments in mice [25]. Systemic injection of recombinant AAV serotype 9 resulted in ChR2 expression in ~10% of the intralaryngeal muscle fibers, which enabled transient light-mediated opening of the vocal cords [25]. Improving expression levels is a crucial goal for this approach to become a feasible therapy. Local injection of the AAV may help achieve higher expression levels [22].

Expression of ChR2 in peripheral motor nerves following local injection of AAV into specific muscle groups has been demonstrated in rats [88, 89, 148] and more recently, in non-human primates [156]. Although these studies showcase the feasibility of AAV gene delivery to enable indirect optogenetic stimulation, there are several hurdles that still need to be overcome.

A general obstacle in the field of AAV-based gene transfer is the presence of neutralizing antibodies, which may significantly lower the efficiency of gene transfer. The chance of an AAV infection accumulates over the life span leading to an immune response and the presence of neutralizing antibodies against several AAV serotypes [150]. These antibodies also neutralize the recombinant AAV, thus significantly reducing the transduction rate. It is well established that neutralizing antibody titer varies greatly from individual to individual [116] and it must be taken into account both in a preclinical and a future, clinical setting. Importantly, different species also show a high variance in neutralizing antibody levels against different serotypes [116], including AAV2, AAV6 and AAV9, which are most commonly used for muscle transduction [153]. For example, rats and mice possess a very low to undetectable quantity of antibodies to these serotypes [116]. This lowers the predictive value of murine models for AAV-based optogenetic therapy in humans. Moreover, most experiments carried out in rodents are performed at a very young age when the immune system has not fully matured yet. In comparison, patients included in one of the biggest clinical trials using AAV gene therapy were on average 60 years old, and close to 60% of participants had to be excluded from the cohort because of too high neutralizing antibody titers [46]. Performing plasmapheresis to get rid of the neutralizing antibodies or transient pharmacological immunosuppression may enhance transduction efficiency and thus allow more patients to benefit from AAV-based optogenetic therapies.

Furthermore, viral dosages have to be increased by several orders of magnitude in larger animals and humans. Results from recent non-human primate studies support this hypothesis [155, 156]. Upon intramuscular injection of hindlimbs and forelimbs of three rhesus macaques, the authors observed a variable expression time-course, dispersed “hotspots” of light-responsive muscle tissue and no functional limb movement. Unfortunately, neutralizing antibody titers were not examined, despite their potential contribution to the expression profiles observed [29, 54]. It is also important to note that AAV-based gene therapy leads to the production of antibodies against the specific serotype of AAV utilized, preventing the re-administration of the same AAV vector [150]. There are only a few AAV serotypes showing adequate tropism towards skeletal muscle tissue, making repeated transductions challenging. It is known that tissue tropism is related to capsid structure. In a recent proof-of-concept study, Ogden et al. (2019) have shown that machine-guided protein engineering can lead to increased tropism towards the desired tissue. Engineering of AAV subtypes with increased skeletal muscle tropism may be a feasible future goal. Moreover, the authors have also identified certain viral capsid mutations that aid escape from antibody neutralization [108]. Evasion of the humoral component of the immune response may in itself improve expression levels. However, further aspects of the immune response to AAV-mediated ChR2 expression also have to be taken into account for the feasibility in humans. Ideally, stable, long-term optogenetic protein expression following one AAV gene transfer should be achieved to allow progression to the clinic. However, this may pose a greater challenge than initially expected.

### Immune response

Achievement of sustained optogenetic protein expression is crucial for optogenetic restoration of skeletal muscle function in humans. Few studies attempted to examine the length of optogenetic protein expression in skeletal muscles and the peripheral nervous system following AAV gene transfer. Importantly, the immune response can be targeted against the AAV as well as against ChR itself and these have to be thus considered separately.

Immune response to AAV-mediated gene therapy is a well-known limitation and has been observed in several animal models, as well as in clinical trials [99]. Neutralizing antibodies lowering transduction efficiency is only the first out of many processes influencing AAV-mediated optogenetic protein expression [150]. Inflammation, followed by an adaptive immune response may lead to the eventual rejection of transduced cells [118]. Anti-AAV T-cell responses have been reported in clinical trials. For example, an AAV capsid-specific cellular response was observed during gene transfers targeting the liver in clinical trials for hemophilia B, which lead to eventual loss of transgene expression [90, 106]. This could be partially rescued by high doses of steroids [106]. Importantly, an AAV-specific, class I major histocompatibility complex-mediated response only lasts as long as the AAV capsids persist within transduced cells, meaning that steroid-based or other forms of acute immunosuppression may resolve this issue.

However, ChR2 and other ChR are expected to be recognized by mammalian immune systems as well as they predominantly originate from phylogenetically distinct species. Conversely, several studies reported that in rats, retinal expression of ChR2 upon AAV gene transfer following local injection does not lead to clinically relevant immunogenic responses [37, 146]. Furthermore, based on regulatory T-cell response, Sugano et al. (2011) hypothesized that the inflammation-like immune reaction observed was caused by AAV and not by ChR2. Although they did detect ChR2-specific antibodies, the titer of these was extremely low. However, due to the immune-privileged nature of the eye, these results do not allow for any conclusions to be drawn regarding AAV-mediated optogenetic protein expression in other tissues.

Maimon et al. (2018) were the first to characterize the immune response following AAV-mediated ChR2 expression in peripheral tissues of rats. The researchers present a compelling argument for the role of ChR2 immunogenicity in optogenetic protein expression loss. Intramuscular injection of AAV carrying the ChR2 gene under a neuron-specific promoter either in fusion to the fluorescent reporter enhanced yellow fluorescent protein or alone appeared equally immunogenic. The same loss of expression time course was observed in both cases, with all rats except one losing expression by 10 weeks post-transduction. Moreover, significant denervation atrophy was observed with the transduced muscle tissue. An immune infiltrate containing elevated CD8+ T cells and CD68+ macrophages adjacent to the transduced motor neurons was seen and anti-ChR2 antibodies were detected in rat sera. Optogenetic expression was extended in immunocompromised rats and immunosuppressive treatment with tacrolimus could significantly extend expression longevity in wild type rats. Importantly, when the human synapsin 1 gene promoter was used, muscle tissue was not an immune target. In contrast when the CAG promoter was tested, which does not restrict expression to neurons, a corroborated immune response was observed and histochemical examination suggested a myocytic immune attack. Furthermore, a high level of mortality (60%) was observed in these rats. However, since the CAG promoter may lead to transgene expression in antigen-presenting and immune cells, it is possible that this contributed to the corroborated immune response [118]. In the future, constructs with muscle-specific promoters should be tested and the immune response following transduction with these characterized.

As mentioned above, AAV-mediated optogenetic protein expression was recently demonstrated in non-human primates [156]. A variable time course of expression was observed in motoneurons of forelimbs and hindlimbs of the animals, with loss of optical sensitivity occurring between 8 and 13 weeks after transduction [156]. The concurrent loss of reporter fluorescence confirmed the temporal loss of expression of both ChR2 and Chronos, a ChR with fast on and off kinetics [68]. Although the authors of this study did not examine the underlying cause, it is probable that the immune response plays an important role in such optogenetic protein expression loss over time. Of note, a study by the same research group reports ChR2 expression in the peripheral nerves of a rhesus macaque for as long as 24 weeks post-transduction [155]. Importantly, the monkey was receiving the immunosuppressant cyclosporine daily until 19 weeks after AAV injection. The loss of ChR2 expression by 24 weeks is likely related to the cessation of immunosuppression.

Importantly, transgene-specific immune responses following AAV gene transfer appear to be highly dependent on the target tissue and the route of virus administration. While AAV vector delivery to the liver seems to induce transgene tolerance in many cases [91, 100, 167], prolonged transgene expression following intramuscular AAV delivery required immunosuppression in several preclinical trials [42, 48, 51, 154]. Furthermore, intramuscular administration of an AAV vector encoding micro-dystrophin to Duchenne muscular dystrophy patients also led to an immune response against the transgene [93]. Notably, Duchenne muscular dystrophy is characterized by chronic inflammation in the muscle tissue, which may provide an additional pro-inflammatory stimulus. This may lead to a corroborated immune reaction to an otherwise low immunogenic gene transfer. Taking all this into account, it is possible that chronic immune suppression is a prerequisite for maintaining ChR expression in skeletal muscle tissue or motor neurons. The cost-benefit ratio of such a drastic, potentially lifelong intervention may deter both clinicians and patients from considering optogenetic interventions as a therapeutic option. Future studies should aim to determine if initial, transient immunosuppression may be sufficient to promote transgene tolerance and prolonged ChR expression.

Interestingly, fluorescent protein immunogenicity has been reported as well [4]. Therefore, it is possible that the reporter corroborates the immune response or is the trigger itself. Although Maimon et al. (2018) observed equal immune activity in ChR2- and ChR2-YFP-transduced rats, in the absence of the reporter, yellow fluorescent portein, the anti-ChR2 antibody titer was significantly lower, which indicates that ChR2 may not be the only trigger for the immune response. Furthermore, though most studies employed the H134R variant of ChR2, other ChR from different species may not only exhibit beneficial biophysical properties but could also affect cellular behavior differently [118] and also trigger a different immune response. Alternatively, ChR with low immunogenicity could be engineered or immune checkpoint molecules such as PD-L1/2 could be included in the AAV to mitigate the adaptive immune response elicited by the transgenic construct [88].

### Optical implants

As transdermal illumination from external sources is technically challenging and would most likely be unpopular among patients, illumination for therapeutic approaches requires the design of adequate optical implants. The technical specifications of such implantable devices, as well as the challenges they face, vary greatly based on the size and nature of the tissue to be stimulated. Hence, indirect and direct optogenetic stimulation of skeletal muscle tissue require distinct optical implants.

In principle, implantable optical devices can be either based on integrated LEDs in close proximity to the target tissue or optical waveguides interfacing a more distant optical pulse generator. While the LED-based variant comprises an electrical implantable pulse generator (IPG) and a flexible cable similar to electrically active implantable medical devices (AIMDs), light sources, such as laser diodes (LDs), will be implemented directly in the IPG in the case of the waveguide-based systems. The light of these LDs will be guided to the tissue of interest, preferably using flexible, polymer-based waveguides. Ideally, the optical implant and its electrical or optical (i.e., waveguide) cables should be detachable from the respective IPG. In this context, LED-based systems are advantageous, as established connector solutions are applicable [73]. On the other hand, a light source hermetically encapsulated in the pulse generator might provide an improved long-term stability in contrast to LEDs encapsulated on a flexible substrate. Moreover, the housing of the pulse generator serves with its larger surface area as an efficient heat spreader for the integrated optoelectronic components, i.e., LDs. In contrast, the integrated LED chips or thin-film micro LEDs (μLEDs) act as localized heat sources in direct contact with the stimulated nerve or muscle. Consequently, specific stimulation parameters are necessary to maintain a temperature increase below 1 K [165].

#### Optical implants for indirect optogenetic stimulation

The key advantage of the indirect optogenetic stimulation of a muscle is that the illumination can be performed at any site of the peripheral nerve, for example, in a region with minor movements of the surrounding tissue and next to bones enabling a proper implant fixation. In addition, peripheral motor nerves are typically not bigger than a few millimeters. Thus, full light penetration of the nerve tissue can be achieved at considerable light intensities even with blue light. Hence, a potential oAIMD used to stimulate peripheral nerves could be similar to a cuff electrode and would face the same challenges in surgery, fixation at the nerve, and its interconnection to the pulse generator [113]. Instead of electrodes, μLEDs or LED chips will be integrated into a bendable, polymeric substrate wrapped around the nerve (Fig. 1a). Dependent on the diameter of the nerve to be stimulated and its optical transmittance, the integrated light sources can either be integrated along the nerve perimeter, or aligned as a linear array along the nerve. With respect to typical nerve diameters on the order of 1 mm and resulting bending radii of the optical cuffs of 0.5 mm, it is obvious that μLEDs with application specific dimensions [69] are superior compared with larger LED chips [129]. Reflective coatings might be implemented in the polymeric substrate similar to the design employed in a recent study where an optical cuff comprised of an optical glass fiber was successfully applied in freely moving mice [96]. Such reflective coatings would circumvent a potential light loss towards the surrounding tissue or neighboring nerves. However, it has to be taken into account that the light stimulus of an optical cuff implant cannot be restricted to individual fascicles controlling single muscles.

In principle, μLED-based optical implants might also be inserted through the nerve (Fig. 1b) similarly to a transverse intrafascicular multichannel electrode arrays used for FES [20]. In this case, arrays of closely spaced μLEDs arranged along a slender probe shaft similar to optical cochlear implants [43, 69] could be inserted and positioned within the nerve using a suture loop [20, 110]. Individual μLEDs (lateral dimensions of 60 × 60 μm^2^, successfully demonstrated by Klein et al. [69] of the high-density array will allow to restrict the optical stimulus to smaller sections of the nerve, thus potentially targeting individual fascicles. However, applying such a transverse intrafascicular optical probe (TIOP) has the risk to disrupt the nerve leading to a loss of function which could have severe impacts on patients’ life quality.

#### Optical implants for direct optogenetic stimulation

Direct optical stimulation of skeletal muscles requires the illumination of the whole muscle. This means that the illuminated area must be as global as possible and light has to efficiently penetrate through all layers, as muscle fibers are electrically insulated from each other and only span approximately two-thirds of the whole muscle tissue. Thus, the use of red light–sensitive ChR would be highly beneficial especially for bigger skeletal muscles due to the increased penetration depth of longer wavelengths compared with blue light of 470 nm used with the conventional ChR2. In contrast, blue light has a short absorption length in the order of 500 μm in muscle tissue [165].

In addition, utilizing red-shifted ChR would reduce the possible phototoxic effect. Prolonging the wavelength of light utilized in biological experiments from 470 to 590 nm enables the increase of light energy more than 1000 times, as photochemical damage shows an exponential decay in correlation with the wavelength of the applied light [15].

The use of illumination directly of the skeletal muscle fibers provides the advantage that light can be restricted to specific muscle groups, which can thus be selectively stimulated. Global illumination of a muscle can be achieved by using a two-dimensional (2D) array of LEDs operated simultaneously (Fig. 2a). These optical implants might be based on polyimide substrates similar to those used in optical cochlear implants [129] or heart pacing devices [36]. The LED array on the polymeric substrate should be encapsulated in silicone rubber, which provides an effective barrier for humidity. This encapsulation will simultaneously improve the mechanical stability of the implant, while leaving its optical transparency unaffected. In order to increase device stretchability, Kirigami-based structures [102, 103] might be applied for the layout of the polymeric substrates. Alternatively, stretchable metallizations integrated into the silicone substrate are foreseeable as well [75, 98]. In the case of 2D arrays, both LED chips or μLEDs can be used as integrated light sources. Since the implants do not need to be bent the way optical cuffs have to, and since larger areas are presumably targeted, LED chips represent a more appropriate solution than μLEDs.

The waveguide equivalent of the 2D LED array is a polymeric substrate with integrated waveguides (Fig. 2b) which comprise mirror structures reflecting light in the direction normal to the plane of the optical probe (Fig. 2c). In order to increase the surface area being optically stimulated with this implant, waveguides might be equipped with multiple mirrors, each reflecting only a portion of the total optical power transmitted through a waveguide [38]. To minimize the size and complexity of the detachable optical connector, a larger waveguide is foreseen that is split into multiple smaller waveguides on the optical implant using Y-splitters (Fig. 2b). In addition, the polymer material might be equipped with a surface of defined roughness, serving as a forward stray layer. The key question here is whether sufficient light intensities can be achieved across the surface of bigger skeletal muscles.

#### Optical implants for therapeutic application

Regardless of the chosen implant architecture (i.e., optical cuff or 2D arrays combined either with LEDs or waveguides), and whether the goal is indirect or direct stimulation, there are several key aspects and challenges that need to be taken into account in order to develop functional oAIMDs for clinical applications. These include small implant dimensions, a high level of system integrity, long-term stability, and the possibility to enable laterally stable stimulation patterns either restricted to a single nerve or addressing a larger, defined surface area of a muscle.

Importantly, the oAIMD needs to be small enough to minimize tissue trauma of the nerve or muscle to be stimulated. At the same time, the implants need to be mechanically robust during surgical interventions and must withstand movements of the muscle itself and the surrounding tissue. Considering approved AIMDs for instance for epilepsy diagnostics using ECoG arrays realized as silicone foils with integrated platinum electrodes [77], as well as micromachined transverse intrafascicular multichannel electrode implants [20] or cuff electrodes [113], we are confident that LED-based optical implants used as 2D arrays, TIOP devices or optical cuffs are technically feasible. Similarly, waveguide-based implants have successfully been demonstrated in the form of a glass fiber–based optical cuff [96]. In this case, polymeric waveguides with an improved bendability and stretchability are expected to improve the biocompatibility of these devices. A recent study suggests that the long-term stability of silicone rubber as a waveguide material is a key prerequisite to enable these optical tools—either as cuffs or 2D waveguide arrays [3].

In order to keep tissue trauma to a minimum, heat generation of integrated optoelectronic components such as LDs and LEDs must be carefully considered and controlled. Light sources integrated into the IPG are beneficial as heat can be efficiently dissipated across the large surface area of the pulse generator. In contrast, LEDs in direct contact with tissue, separated by the encapsulating material only, will potentially cause tissue heating. However, as demonstrated recently, LED chips used to optically stimulate an isolated heart can be operated by appropriately chosen LED currents and duty cycle values such that the temperature increase remains well below 1 K, while enough optical power for cardiac pacing is provided [165]. Furthermore, in order to achieve long-term stability of the optical implants, their encapsulation against body fluids is of utmost importance. Preferably, the encapsulation should be hermetic which is however difficult to achieve in the case of polymeric materials. To date, only metallic housings with respective feedthrough—either electrical or optical—as used in clinically applied AIMDs, fulfill the most stringent requirements. In the case of LEDs integrated into flexible polymeric substrates, attempts with a combination of fluoropolymers and silicone rubber have been made [13, 129] which still require improvements. Multilayer stacks based on Parylene C or polyimide, and atomic layer deposited films of Al_2_O_3_, HfO_2_ and TiO_2_ have demonstrated superior performance to encapsulate microelectronics implemented in flexible implants [95, 125, 151] and thus provide a promising alternative.

While cuff or TIOP implants stimulating peripheral nerves might benefit from the restriction in the optically stimulated volume, implants for direct skeletal muscle activation might require increased emittances to illuminate a sufficiently large volume of muscle tissue. However, increased emittance is attributed to higher LED currents resulting in increased tissue heating [11]. LEDs operating at longer wavelengths with an improved penetration depth are available as housed systems pigtailed to optical waveguides (e.g., from PlexonPlexon, Dallas, TX, USA), or small LED chips. In the case of μLEDs, only a limited number of technical solutions operating at longer wavelengths have been published so far [67]. The further improvement of these solutions is an important goal given the increased light penetration into tissue at longer wavelengths and safety thresholds.

The implantable pulse generators for LED-based oAIMDs are essentially identical to IPGs with established electrical feedthroughs and detachable connectors [73]. For implants using optical waveguides, integrated solutions will use optical feedthroughs [64]. They might be based on housing-integrated optical glass fibers used as an integral part of the implant or the glass fibers can be cleaved with the silicone rubber-based waveguide directly attached to short fiber sections. Alternatively, fiber ferrules can be applied which are part of a detachable optical connector.

#### Succesfull examples for oAIMDs

The widespread experience gained with implantable medical devices designed for electrical stimulation, as well as the first tools for basic optogenetic research are an important source of inspiration for the design of oAIMDs. Over the past decades, a widespread variety of clinically approved, electrically active medical implants for the diagnosis and treatment of different diseases have been developed. Starting from fully implantable cardiac pacemakers in 1958 [76], these implants are nowadays also utilized in the central and peripheral nervous systems restoring sensory functions such as hearing (Cochlear [164]) and auditory brainstem implants [33]), vision (Retina implant [133]), as well as motor functions (FES to control a limb prosthesis [61]) and the possibility to provide sensory feedback [110]. In addition, these electrical implants are used for deep brain stimulation in Parkinson’s patients to reduce tremor [40] and address psychiatric disorders such as depression [128], and to treat chronic pain [52, 101]. In general, these electrical AIMDs comprise (i) a set of stimulating electrodes to interface the targeted tissue, (ii) an IPG which contains the electronic control and a battery, and (iii) cables to interconnect electrodes and generator. This requires compact, mechanically robust implants positioned subcutaneously able to withstand excessive forces exerted during device implantation. Most often, pulse generator and electrodes have to be implanted at distances as far as 45 cm. As a representative example, the pulse generator in deep brain stimulation applications is positioned in the subclavicular region connected to the electrodes implanted in the subthalamic nucleus. Preferably, electrodes and generator are connected via detachable systems to improve installation, and facilitate replacement and troubleshooting [73]. Furthermore, in order to avoid a mechanical failure of the interconnecting leads due to a relative movement between generator and electrodes, helically wound cables should be applied, providing a certain degree of stretchability.

Optical implants that have been developed so far are mostly utilized in basic research, either in vitro or in vivo using small animal models. Their initial application as intracortical implants required a pronounced miniaturization in order to reduce tissue trauma. Despite the possibility for using external light sources, optical glass fibers applied in vivo in first optogenetic studies [140, 159] have certain disadvantages. These include an increased bending stiffness, the need for interconnecting ferrules with diameters above 1.5 mm, limiting the number of fibers applicable per subject, and the fact that each fiber represents a single stimulation site only. The latter disadvantage is circumvented by the use of tapered glass fibers covered by an opaque metal layer which comprises small optical windows realized by focused ion beam processing [112]. These windows can be independently addressed by changing the angle of incidence under which light is coupled into these fibers, addressing different depths inside the tissue [111]. Furthermore, optical fibers have been combined with silicon based electrode arrays, enabling the simultaneous optical stimulation and electrophysiological recording from the tissue area of interest [119, 137]. In order to further reduce the implant size, silicon-based, penetrating tools have been equipped with microfabricated optical waveguides based on polymers, or silicon-nitride and silicon-oxide used as waveguide core and cladding materials [132], respectively. Implementing nanophotonic structures for wavelength division multiplexing a single fiber can be used to address different emitters arranged along a single slender shaft, minimizing the number of fibers per implanted system [132, 135]. Depth control of tissue illumination is achieved by tuning the wavelength of the external light source.

Compact, fibreless, electrically controlled optical implants have been created with small LDs implemented in silicon-based optical probes, comprising integrated waveguides [63, 130]. The LD chips are butt-coupled to the waveguides and encapsulated using micromachined housings [130]. However, each waveguide requires a single LD chip, which might ultimately limit the number of light sources that can be integrated.

An alternative approach to the use of waveguide-based systems is the application of LEDs. These have been successfully utilized in basic optogenetic research either as LED chips with lateral dimensions in the range of several 100 μm and thicknesses above 50 μm [12, 60, 65, 165], or as μLEDs with a thickness of ca. 5 μm and custom-designed dimensions as small as 5×5 μm^2^ [11, 43, 67, 69, 117, 124, 157]. In LED chip-based systems, the LEDs are soldered or flip-chip bonded to flexible substrates. They can either be used as a single light source [158] or as 2D LED arrays on tissue surfaces such as the brain [65], heart [36], or as a linear LED arrangement in the curved geometry of a cochlea [129]. Stiffened structures facilitate the optical probe insertion into cortical [12] or cardiac tissue [165] enabling a depth-dependent optical stimulation. Implants based on μLEDs either apply stiff silicon substrates [11, 66, 124, 157] or polymers [43, 47, 67, 69] with the gallium nitride layer stack of the μLED either being epitactically grown on silicon substrates [124, 157] or transferred from a sapphire substrate using wafer bonding and laser lift-off [43, 69, 70] or transfer printing [67, 136]. The devices are applied in basic neuroscientific research to investigate brain functions [47, 66, 67, 124, 157] or in translational studies, for example as an optical cochlear implant [43, 69].

### Specific pathophysiological aspects for translation of optogenetic stimulation

Given the above described caveats and requirements of optogenetic approaches, its use and translational perspective has to be carefully considered. The future clinical application of these approaches is expected to be limited to cases when effective alternatives do not exist or suffer from severe side effects. Furthermore, it is important to evaluate carefully whether direct or indirect optogenetic stimulation appears more appropriate for each case. We will discuss this exemplarily with four diseases showing the ascending complexity of the underlying neuromuscular systems.

FES of the phrenic nerve to restore normal ventilation via diaphragm contraction was tested in a clinical study. With this approach, patients could be weaned from machine ventilation after bilateral lung transplantation [147]. In general, phrenic nerve stimulation is a good example for indirect stimulation, as the motoric part only innervates the diaphragm and breathing is a binary task consisting of contraction and relaxation only. Since the phrenic nerve also consists of afferent sensory neurons, FES can result in shoulder pain at the respective ‘Head-Zones’ [147]. Thus, indirect optogenetic stimulation with selective expression of ChR2 in the motoric nerve fibers may pose a better alternative. On the oher hand, direct optogenetic stimulation of the diaphragm will be technically challenging, as the whole muscle would have to be illuminated requiring excessive surgical procedure and a significantly higher energy demand.

In contrast, the human larynx consists of several small muscle groups in close proximity. Here, the recurrent laryngeal nerve innervates all skeletal muscles involved in opening the vocal folds to allow air passage during breathing, and in closing for phonation and protection from aspiration. The most common cause of recurrent nerve paralysis is iatrogenic, namely thyroid surgery [16]. Paralysis of the recurrent nerve is in one quarter bilateral and results in a fixed paramedian position of the vocal folds and eventually life-threatening dyspnea. Established treatment options consist of a multitude of surgical interventions, aiming to restore respiration [10, 80], while having to accept impaired phonation and slightly higher risk of aspiration. On the other hand, a permanent tracheostomy, which bypasses the glottic stenosis, is associated with adequate results in terms of voice and phonation but leads to stigmatization and therefore has a severe impact on the patients’ quality of life. Selective re-innervation of the bilateral posterior cricoarytenoid muscles was studied intensively [34]. Unpleasant side effects due to laryngeal synkinesis were reported. Furthermore, electrical stimulation of the posterior cricoarytenoid muscle has been explored since the 1970s in various animal species, as well as in patients. While opening of the vocal folds is in principle possible [31, 41, 104, 114, 160–162], severe side-effects prevent its routine clinical application. These side-effects include corrosion or encapsulation of the electrode tip reducing the efficiency of stimulation, discomfort due to the activation of sensory nerves, and co-stimulation of antagonistic muscles, which in some patients requires the selective silencing of antagonistically acting muscle groups by repetitive botulinum toxin injections [160, 161]. Indirect optogenetic stimulation of the larynx could circumvent some of the aforementioned disadvantages, for example the uncomfortable sensations caused by FES. On the other hand, the synkinetic innervation of antagonistic muscles will limit its utility. Therefore, the direct stimulation of the intralaryngeal muscles is a good example for direct optogenetic stimulation. Direct optogenetic stimulation in a mouse model was able to selectively close and open the glottis as the high spatial resolution allowed specific illumination of separate intralaryngeal muscles [25]. Selective expression in intralaryngeal muscles will circumvent the activation of sensory receptors and antagonistic muscles. In this setting, where repetitive long-term stimulation is necessary, selective expression in slow muscle fiber types and thus orderly recruitment of motor units is expected to prevent fatigue.

A further even more complex example is the facial nerve. The facial mimic presents one of the most complex muscle interplays in humans. Patients with complete facial nerve paresis suffer from impaired lid-closure, as well as oral insufficiency, which are devastating due to a loss of function, as well as cosmetic consequences [2]. In most cases, the cause of the facial paresis lies in the peripheral nerve itself, which is often idiopathic, a complication of surgeries or the result of infections [166]. The respective underlying pathomechanism determines which way to restore muscle function is most likely beneficial for the patients.

Surgeries could affect the facial nerve from the brainstem’s nucleus down to the parotid region. Normally the leftover nerve trunk is intact. Therefore, restoration via FES is feasible [62] but does neither prevent stimulation of synkinetic muscles, nor that of sensory fibers, which is problematic in this highly sensitive area. In comparison, indirect optogenetic stimulation could circumvent uncomfortable sensations [74]. As described above, indirect optogenetic stimulation could restore the function of two different muscle groups with two different ChR but is unable to restore the complex interplay of the myriad of mimic muscles.

Several infectious diseases can affect the facial nerve, most commonly varicella-zoster virus [58], leading to the devastation of the facial nerve. Antiviral and steroidal therapeutics are routinely administered, but paresis persists in some patients. Direct stimulation could restore the vital function, as well as improve the cosmetic aspects. In a recent study, transdermal electrical stimulation above the required muscle was performed in healthy participants [55]. Visually observable movement induced by forehead and lower lip muscles was noted. The authors recommend an electromyographic input from the contralateral healthy site in the sense of open-loop stimulation [55, 162]. However, in some participants painful sensations were observed. Furthermore, transcutaneous stimulation targets the synaptic junction and not directly the muscles themselves, and therefore fails when the motoric junction is unfunctional (e.g., myasthenia gravis). Direct optogenetic stimulation of the muscles may pose a better alternative. Given the complex interplay of several small muscles for the mimic, the high spatial resolution of optogenetic stimulation could be beneficial. However, transcutaneous optogenetic stimulation would require optical devices right in front of the face. This would likely impair patients’ quality of life, since the illumination is within the visible wavelength region. Furthermore, it has a high energy demand, or would require high light sensitivity. Therefore, a more feasible approach is to place the illuminating devices right beneath the muscles with the caveat of required surgery.

To demonstrate its far-reaching applicability, direct optogenetic stimulation of the external urethral sphincter can be considered. This muscle provides the arbitrary retention of urine and is located within the pelvic diaphragm. A recent study showed no improvement within a non-invasive intravaginal electrical stimulation for stress-urinary-incontinence in women [144]. Since the pudendal nerve innervates the whole pelvic diaphragm, there might be anatomical difficulties to apply indirect stimulation either electrical or optogenetical, whereas the direct optogenetic stimulation could be more likely and perception-free, which is also extremely important in this area. However, the external urethral sphincter would require a permanent stimulation and side effects like thermal damage, as well as light-induced damage under permanent illumination, need further investigation.

To conclude, the decision between direct or indirect optogenetic stimulation should be based on several equally important parameters: (A) the anatomical properties, (B) the complexity of the physiological function, (C) the underlying pathomechanism, and (D) the feasibility of oAIMDs.

## Conclusion

The physiology of skeletal muscles explains why the treatment of paralysis is a complex challenge, where the underlying pathology, as well as the unique anatomical site and physiological function of the affected muscle, has to be considered. Although FES is a viable therapeutic approach in some cases, its limitations prompt a search for alternative therapies. Direct and indirect optogenetic stimulation of skeletal muscle tissue have great potential to become new treatment options in the future. Depending on the site and nature of the pathology, one or the other might be more advantageous. Importantly, clinical progression of these methods will only become possible after successfully establishing gene transfer leading to sustained optogenetic protein expression. Some preliminary data strongly suggest that a potential immune response is a major hurdle in this context [88, 155]; hence, this requires further investigation. Moreover, the proof-of-concept for oAIMDs in relevant clinical setting has to be delivered. Importantly, the clear need for novel treatments aiming to restore skeletal muscle function in patients suffering from conditions such as bilateral laryngeal paralysis, facial nerve paresis and diseases such as amyotrophic lateral sclerosis and myasthenia gravis underlies the importance of research to enable the clinical translation of optogenetic stimulation of skeletal muscles.

## Abbreviations

AAV: Adeno-associated viruses
AIMD: Electrically active implantable medical device
CAG: Chicken-β-actin promotor
ChR: Channelrhodospin variants
ChR2: Channelrhodopsin2
FES: Functional electrical stimulation
IPG: Implantable pulse generator
LD: Laser diode
LED: Light-emitting diodes
oAIMD: Optically active implantable devices
TIOP: Transverse intrafascicular optical probe
2D: Two-dimensional
